# Systematic review and meta-analysis on prevalence, pattern, and factors associated with ocular protection practices among welders in sub-Saharan Africa

**DOI:** 10.3389/fpubh.2024.1397578

**Published:** 2024-06-17

**Authors:** Yibeltal Assefa Atalay, Natnael Atnafu Gebeyehu, Kelemu Abebe Gelaw

**Affiliations:** ^1^School of Public Health, College of Health Science and Medicine, Wolaita Sodo University, Wolaita Sodo, Ethiopia; ^2^School of Midwifery, College of Health Science and Medicine, Wolaita Sodo University, Wolaita Sodo, Ethiopia

**Keywords:** prevalence, ocular protection practice, welders, systematic review, meta-analysis, sub-Saharan Africa

## Abstract

**Background:**

Due to hazardous working conditions, welders are more likely to be exposed to mild to severe eye issues during the welding process. Globally, this issue is a major contributor to vision loss and blindness. One of the most frequent causes of unilateral blindness in the globe is ocular injury.

**Objective:**

This review aimed to assess the pooled prevalence of ocular protection practice and associated factors among welders in sub-Saharan Africa.

**Methods:**

Databases including PubMed, Scopus, web of Science, Google Scholar, and the African Journals Online were systematically searched for relevant literature. The statistical analysis was performed using STATA data analysis software version 14, while Microsoft Excel was used for data abstraction. We checked publication bias using a funnel plot and Egger and Begg regression tests. A *p*-value < 0.05 was considered significant, suggesting the presence of presence publication bias. The I^2^ statistics were used to assess heterogeneity between studies. The study's overall effect was evaluated using the random effects model.

**Results:**

From retrieved 2,326 original studies, 17 studies were included in the final pooled prevalence analysis. The overall prevalence of ocular protection practice among small-scale welders in sub-Saharan Africa was 53.71% (95% CI: 42.54, 64.88). Having pre and in-service training [AOR: 4.97, 95% CI: (2.64, 9.36)], having work experience as a welder [AOR: 4.94, 95% CI: (3.24, 7.54)], and having a history of ocular injury [AOR: 2.99, 95% CI: (1.58, 5.66)] were significantly associated with the ocular protection practices.

**Conclusions:**

In sub-Saharan African countries, the ocular protection practices among small-scale welders were low. Furthermore, the current meta-analysis found ocular protection practice to be significantly associated with on-the-job training, work experience of welders, and a history of ocular injury in the past year of small-scale welders in sub-Saharan Africa. This review will serve as baseline data for further studies to generate inputs for eye care providers and policymakers to improve good practice levels about ocular protection. Policies should be put in place to ensure all welders use proper personal–protective equipment, and receive regular health training.

## Introduction

Welding is an occupation fraught with hazards, as it involves the process of separating a single metal piece into two parts and subsequently reconnecting them using a flame, electric arc, or alternative heat sources. Furthermore, welders persistently encounter substantial occupational health and safety issues, particularly pertaining to ocular health risks associated with prolonged exposure to welding activities (1). Welders are constantly exposed to the risk of eye injuries and diseases through their profession (2). As a result of the hazardous working conditions, welders are more susceptible to mild to severe eye problems during the welding process (3). However, about 90% of occupational injuries are preventable by using appropriate safety devices, and individuals working in hazardous occupations are at high risk (3, 4).

Annually, more than 500,000 ocular injuries leading to blindness take place worldwide, with approximately 1.6 million individuals suffering from blindness and 2.3 million experiencing bilateral visual impairment as a result of eye injuries (5). Research conducted in sub-Saharan African nations has uncovered the prevalence of significant eye injuries within small-scale industries, such as welding (6, 7). Work-related ocular injuries can be significantly reduced by implementing appropriate eye protection measures in various industrial activities, including welding by ensuring the use of adequate and proper eye protection practices, these injuries can be largely prevented (8).

Small and medium-sized industries account for about 80% of the global workforce; however, there is a clear lack of compliance with health and safety regulations (9, 10). Despite having individual ocular protective equipment and being aware of the ocular risks associated with their jobs, not all welders were using ocular protective devices (11). Absorbing infrared rays through a green glass filter is the most important approach to protecting eyes from the harmful effects of welding arc radiation. Strategically placed in the welding helmet, this filter serves the dual purpose of providing additional protection against physical injury and improving overall safety measures (12).

Unfortunately, there exist instances wherein employees fail to utilize safety glasses or goggles owing to various factors, such as underestimating the potential risks, neglecting proper lens maintenance, experiencing discomfort, the necessity to wear corrective lenses underneath, and personal aesthetic considerations (13). Welders' non-compliance is compounded by their limited understanding (knowledge) of the proper use of protective equipment during welding operations (14).

Welders in low- to low-middle-income countries across the globe still face occupational safety hazards due to eye health risks associated with sweating (15). Research conducted in sub-Saharan countries has found that serious eye injuries occur frequently in small-scale welding industries (16, 17). Similarly, a study conducted in Addis Ababa, Ethiopia, showed that an overwhelming 99.6% of welders reported experiencing vision problems during the welding process (18). Welding protection practices refer to the measures taken to safeguard the eye against potential health hazards that may arise from exposure to welding. This objective is accomplished through the utilization of personal safety glasses, which serve as a protective barrier for the eye (19).

However, in Africa, there is evidence that the level of eye protection practice among welders ranges from 2.3% to 96.9% (20). Welders exposed to ultraviolet and infrared radiation, metal fumes, particulate matter, and thermal burns can suffer severe acute eye diseases that can result in impaired vision. Unfortunately, these risks cannot be prevented or reduced by using standard safety glasses. Hence, it is of utmost importance to incorporate efficient tactics that are directed toward enhancing the understanding and utilization of personal protective eyewear by welders during the execution of welding activities. To our knowledge, there is no systematic review and meta-analysis on the prevalence, patterns, and factors associated with eye protection practices among small-scale welders in sub-Saharan Africa. Therefore, this research addresses the existing knowledge gaps by investigating the combined prevalence of ocular protection practices and the factors associated with them among welders in sub-Saharan Africa. Hence, this research will act as fundamental information for future investigations to provide valuable insights for healthcare professionals and policymakers to formulate evidence-based strategies aimed at minimizing the risk of eye injuries in occupational settings. Finally, the main objective of this study was to determine the prevalence of eye protection practices and associated factors among welders in sub-Saharan Africa.

## Methods

### The study protocol registration and reporting

The purpose of this systematic review and meta-analysis is to determine the pooled prevalence, pattern, and factors associated with ocular protection practices in small-scale welders in sub-Saharan Africa. To ensure the accuracy and completeness of the study, the Preferred Reporting Items for Systematic Reviews and Meta-Analyses (PRISMA) 2020 checklist was used (21) (Supplementary material S1).

### Searching strategies and sources of information

Two approaches were followed to search potentially relevant studies. The electronic database search (PubMed, Google Scholar, Hinari, and Institution research repositories) and the manually search of the reference list of the previous prevalence studies were carried out to retrieve more articles. The literature search was limited to studies published in English that examined the pooled prevalence, pattern, and factors associated with ocular protection practices among welders in sub-Saharan Africa. To ensure comprehensive coverage of the literature, the reference lists of the included studies were carefully checked. A systematic approach was followed to conduct an advanced search on PubMed. Initially, search terms were formulated for four key areas: “Ocular protection practices,” “associated factors,” “Welders,” and “Sub-Saharan Africa.” These keywords were retrieved from Google Scholar and then individually searched in PubMed to identify relevant MeSH terms within the MeSH hierarchy tree. These terms were then combined using advanced Boolean search logic, specifically using the “AND” and “OR” operators to effectively bring the concepts together. The search was also made by combining the above search terms with the names of all countries included in sub-Saharan Africa. The search period was from July 1/2023 to August 10/2023. The three investigators (YAA, KAG, and NAG) independently and carefully reviewed the contents of each retrieved articles. Those literatures fulfilling the following criteria were finally included in the review.

### Population, exposure, context and outcomes frameworks

Population: Welders. Exposure: Exposure is a key factor that increases or decreases the likelihood of Ocular protection practice among small-scale industry welders in Sub-Saharan Africa. Context: Sub-Saharan Africa. Condition: Ocular protection practices. Outcome measurement: The primary outcome of the study was the prevalence of ocular protection practices among welder workers in small-scale industries in sub-Saharan Africa. The secondary outcome of the study was determinants influencing the primary outcomes.

### Inclusion and exclusion criteria

The present study included primary research studies that reported on the dissemination of pooled prevalence, pattern, and factors associated with ocular protection practices among welders in sub-Saharan Africa. The review included full-text articles in English as well as primary studies involving welders. Both published and unpublished articles were considered. However, studies without full-text access and articles that did not provide sufficient information on the outcomes of interest (ocular protection practices) were excluded. In addition, studies were not freely available in full text and those that used a qualitative study design were excluded from the review.

### Data extraction process

Three authors (YAA, KAG, and NAG) working independently abstracted the relevant data from studies by using a standardized Microsoft Excel spreadsheet. The JBI tool for prevalence studies (22) was used as a guideline for data extraction from the finally selected articles. The data extraction tool contains information on the author and year of the study, title, year study was conducted and year of publication, study area and country, sub-region, study design and type, sample size, response rate, the outcome measured, and prevalence rate. Information regarding the publication status was also collected. Additionally, the tool contains information on the study quality scores.

### Critical appraisal and reliability checkup

After screening the relevant studies, the selected studies were critically appraised for methodological validity using Joanna Briggs Institute (JBI) appraisal tools for prevalence studies (22). The tool had a total of 8 questions (Q1–Q8) and those studies with positive answer of more than 50% of the tool (i.e. “Yes” for 5 or more question of JBI tool) were included for this meta- analysis. These scoring was done by two investigators (YAA and KAG) with the discrepancies were resolved with discussion and consensus.

When the disagreement between the two authors was not resolved with discussion, the third author (NAG) involved was as breaker. During critical appraisal of each primary study, more emphasis was given to the appropriateness of the study objectives, study design, sampling technique, data collection technique, statistical analysis, any sources of bias, and its management technique. The parameters are about the following questions: Where were the criteria for inclusion in the sample clearly defined? Were the study subjects and, therefore, the setting described in detail? Was the exposure measured result validly and reliably? Were the main objective and standard criteria used to measure the event? Where confounding factors identified? Were strategies to affect confounding factors stated? Were the results measured indeed and dependably? And, was the statistical analysis suitable? (Supplementary material S2).

### Operational definition

Small-scale industry: Industries with fewer than 10 employees that use power-driven machines.

Ocular protection practice: Participants who scored at the mean and above were considered to have good eye protection practices, while participants who scored below the mean were considered to have poor eye protection practices. Welder: People who carry out welding or other work in welding workshops. Small-scale welding: places like welding houses, garages, and furniture. Ocular injury: Any injury occurring to the eye and or adnexa that occurred in the participant's regular place of work during working hours in the past year.

### Data analysis

The data analysis software Stata version 14 was utilized to perform the statistical analysis. The pooled prevalence estimation was performed using the random effects technique, the foundation of which is the inverse variance method. The evaluation of heterogeneity among research studies was conducted by computing the I^2^ statistic. This statistical measure is the percentage of total variation among studies that can be attributed to heterogeneity rather than chance.

The levels of heterogeneity were denoted as low, moderate, and high using values of 25%, 50%, and 75%, respectively. Sensitivity analysis with the “leave-one-out” approach was used to evaluate the possible impact of any particular study on the meta-analysis. Additional analyses were conducted by considering subgroups to explore potential sources of heterogeneity. The researchers employed Egger's linear regression test and funnel plot examination to investigate potential publication bias. A 95% confidence interval accompanied the measured-adjusted odds ratio.

## Results

### Search and study selection

**Figure 1:**
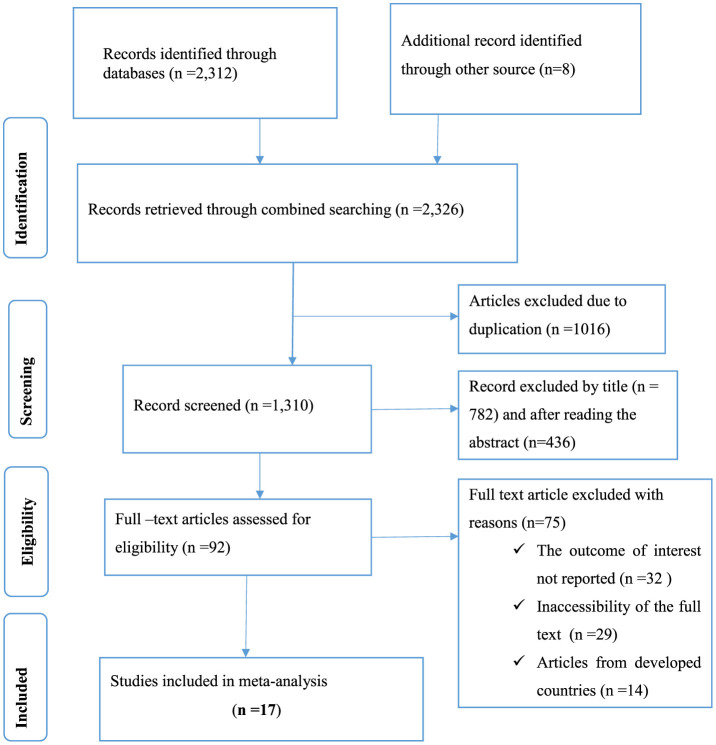
PRISMA flow diagram explaining selection of primary studies in sub-Saharan Africa.

This systematic review and meta-analysis included published studies conducted on prevalence, pattern, and factors associated with ocular protection practices among welders in sub-Saharan Africa. A total of 2,326 records were retrieved through electronic database searching. From these, 1,016 duplicated records were excluded, and from 1,310 articles screened using their titles and abstracts, 1,218 were excluded. Ninety-two full-text articles were assessed for eligibility. From these, 75 full-text articles were excluded from prior criteria, and finally, 17 full-text primary articles were selected for quantitative analysis (Figure 1).

### Study characteristics

Seventeen primary studies (6, 14, 23–37) involving 4,496 participants were included in the systematic review and meta-analysis, and all studies were published from 2003 to 2023. The sample size ranged from 58 to 433. Seven studies were conducted in Nigeria (14, 28, 30–32, 34, 35), two studies in Ghana (6, 29), two studies in South Africa (24, 36), two studies in Ethiopia (27, 33), two studies in Uganda (25, 26), one study in Kenya (23) and, one study in Tanzania (37). The risk level of each study was assessed and we found that all studies were rated as low risk of bias. Regarding the study design, all studies used a cross-sectional study design. Furthermore, the primary studies were categorized according to their sub-regions, six studies were conducted in East Africa (23, 25–27, 33, 37), nine studies were in West African countries (6, 14, 28–32, 34, 35), two studies were in South African countries (24, 36) (Table 1).

**Table 1:** The characteristics of the studies included in the systematic review and meta-analysis.

**References**	**Pub. Year**	**Country**	**Study design**	**Sample size**	**POPP**	**Study quality**
Yego and Ragot (23)	2020	Kenya	CS	100	48.45	Low risk
Sithole et al. (24)	2009	South Africa	CS	150	89	Low risk
Atukunda et al. (25)	2019	Uganda	CS	343	59.9	Low risk
Kwaku et al. (6)	2020	Ghana	CS	382	47.9	Low risk
Ajayi et al. (14)	2011	Nigeria	CS	405	83	Low risk
Itiakorit et al. (26)	2021	Uganda	CS	327	87.8	Low risk
Belete et al. (27)	2023	Ethiopia	CS	396	56.8	Low risk
Eze et al. (28)	2015	Nigeria	CS	343	34.9	Low risk
Abu et al. (29)	2016	Ghana	CS	433	39.5	Low risk
Mary et al. (30)	2020	Nigeria	CS	260	34.2	Low risk
Osagiede et al. (31)	2020	Nigeria	CS	58	46.6	Low risk
Ezinne et al. (32)	2021	Nigeria	CS	230	17.7	Low risk
Belete et al. (33)	2021	Ethiopia	CS	430	40.23	Low risk
Aziegbe et al. (34)	2021	Nigeria	CS	103	60.2	Low risk
Ifeanyi et al. (35)	2022	Nigeria	CS	101	42.91	Low risk
Xulu-Kasaba et al. (36)	2021	South Africa	CS	120	73	Low risk
Rongo et al. (37)	2003	Tanzania	CS	315	50.25	Low risk

CS, Cross-sectional; POPP, Prevalence of ocular protection practices.

### Prevalence of ocular protection practice among welders in sub-Saharan Africa

**Figure 2:**
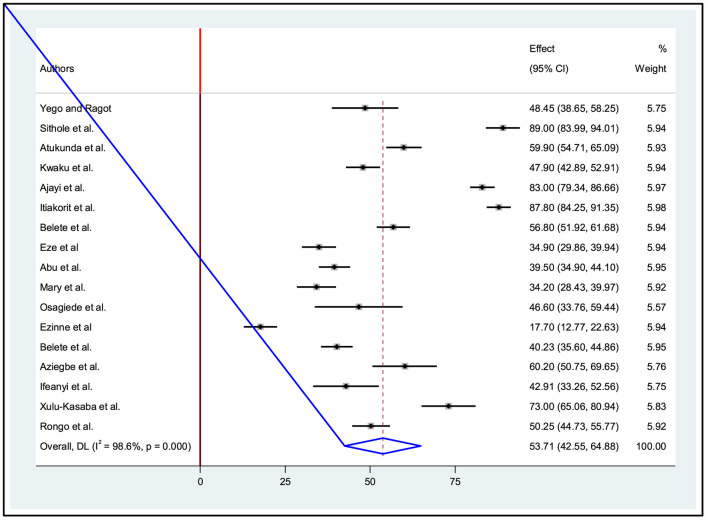
Forest plot for good ocular protection practices among welders in sub-Saharan Africa.

A comprehensive analysis was conducted on a sample of 4,496 participants to determine their level of good ocular protection practice among welders in small-scale industries. The study included a total of seventeen research studies from this, six in East Africa, nine studies in West Africa, and two studies in Southern African countries. The overall pooled prevalence level of ocular protection practice in sub-Saharan Africa was 53.71% (95% CI: 42.54, 64.88). It was determined using the random-effect model. This estimate was statistically significant at a *P*-value *?* 0.001. Furthermore, heterogeneity between studies was found to be high with an I^2^ value of 98.6% (Figure 2).

### Sub-group analysis

To examine the effects of sub-regions, subgroup analysis was conducted. It was found that the study conducted in Southern Africa had the highest combined prevalence of ocular protection practices among welders, reaching 81.30% (95% CI: 65.64, 96.97). Furthermore, I^2^ statistics showed a significant level of heterogeneity (I^2^: 98.6%, *P* < 0.001) between studies. Similarly, the second highest pooled prevalence of ocular protection practice among welders in sub-Saharan Africa was found in Eastern Africa with 57.33% (95% CI: 40.90, 73.76), and I^2^ statistics showed a high degree of heterogeneity (I^2^: 98.4%, *P* < 0.001) between studies. On the other hand, West Africa had the lowest pooled prevalence at 45.18% (95% CI: 29.39, 60.98), and I^2^ statistic showed a high degree of heterogeneity (I^2^: 98.6%, *P* < 0.001) between studies (Table 2).

**Table 2:** Sub-group analysis for the pooled prevalence of ocular protection practices among small-scale welders in sub-Saharan Africa.

**Variable**	**Characteristics**	**D +L pooled estimate with 95% CI**	**Participants**	**No. of studies**	**I^2^ (*p*-value)**
Sub-groups	Eastern Africa	57.33 (40.90,73.76)	1,911	6	98.4% (<0.001)
	Western Africa	45.18 (29.39, 60.98)	2,315	9	98.6% (<0.001)
	Southern Africa	81.30 (65.64, 96.97)	270	2	91.0% (<0.001)
**Total**		**53.71 (42.54, 64.88)**	**4,496**	**17**	**98.6% (<0.001)**

### Sensitivity analysis

In addition to conducting subgroup analyses, we performed a sensitivity analysis by excluding each study to investigate the origin of heterogeneity. This analysis showed that omitting one study had no statistically significant effect on the overall evaluation of the studies (Table 3).

**Table 3:** A sensitivity analysis for the prevalence of ocular protection practice among small-scale welders in sub-Saharan Africa.

**Study omitted**	**Pooled estimate**	**95% conf. interval**
Yego and Ragot (23)	54.03	(42.42, 65.64)
Sithole et al. (24)	51.48	(40.38, 62.59)
Atukunda et al. (25)	53.32	(41.40, 65.24)
Kwaku et al. (6)	54.07	(42.20, 65.95)
Ajayi et al. (14)	51.85	(40.69, 63.01)
Itiakorit et al. (26)	51.55	(40.99, 62.10)
Belete et al. (27)	53.51	(41.54, 65.49)
Eze et al. (28)	54.90	(43.38, 66.41)
Abu et al. (29)	54.61	(42.91, 66.30)
Mary et al. (30)	54.94	(43.43, 66.44)
Osagiede et al. (31)	54.13	(42.58, 65.68)
Ezinne et al. (32)	56.00	(45.49, 66.51)
Belete et al. (33)	54.56	(42.84, 66.28)
Aziegbe et al. (34)	53.31	(41.68, 64.94)
Ifeanyi et al. (35)	54.37	(42.79, 65.95)
Xulu-Kasaba et al. (36)	52.51	(40.90, 64.13)
Rongo et al. (37)	53.93	(42.09, 65.76)
**Combined**	**53.71**	**(42.54, 64.88)**

### Meta-regression analysis

A meta-regression analysis was performed to determine the presence of statistically significant heterogeneity, indicated by an I-squared test statistic of <0.05. The main objective of this analysis was to identify the underlying cause of heterogeneity to ensure an accurate interpretation of the research results. However, the results of the meta-regression analysis did not show any significant variables that could account for the observed heterogeneity. In particular, there were no statistically significant study-level covariates such as sample size or publication year, country, and sub-region that could explain the differences in the level of good eye protection practices among welders. It can be concluded that the heterogeneity observed in this review is likely due to other factors not considered in the analysis (Table 4).

**Table 4:** Meta-regression analysis of factors affecting between-study heterogeneity.

**Source of heterogeneity**	**Coefficient**	**Standard error**	***P*-value**
Year of Publication	−0.021	0.121	0.861
Country	0.093	0.329	0.781
Sample size of the studies	0.001	0.006	0.985
Sub-region	−0.169	0.691	0.809

### Publication bias (reporting bias)

**Figure 3:**
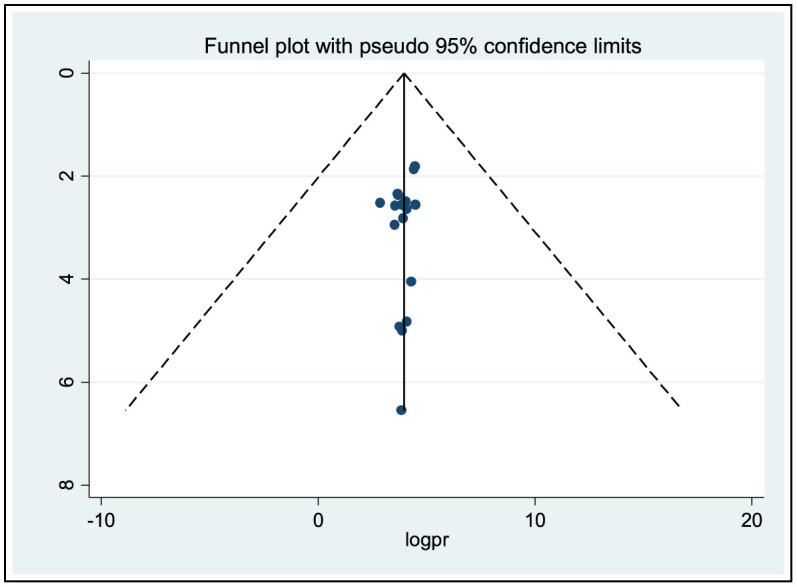
Funnel plots for publication bias of ocular protection practices among small-scale welders in sub-Saharan Africa.

The presence of publication bias was assessed using a funnel plot and the application of the Egger and Begg regression test at a significant level of 5%. The symmetrical arrangement of the included studies, as shown in the funnel diagram, indicated the absence of publication bias. Furthermore, there was no statistical evidence to support the existence of publication bias, and Begg and Egger tests yielded *p*-values of 0.773 and 0.365, respectively, which were not statistically significant. Consequently, the test results provide no evidence of a small study effect (Figure 3).

### Factors associated with ocular protection practice in sub-Saharan Africa

A meta-analysis was conducted using the random effects model to determine the factors associated with ocular protection practices. Therefore, this review found a significant association between the pooled prevalence of ocular protection practice and factors such as training, work experience, and history of workplace eye injuries.

### The association between ocular protection practice and job training of welders

**Figure 4:**
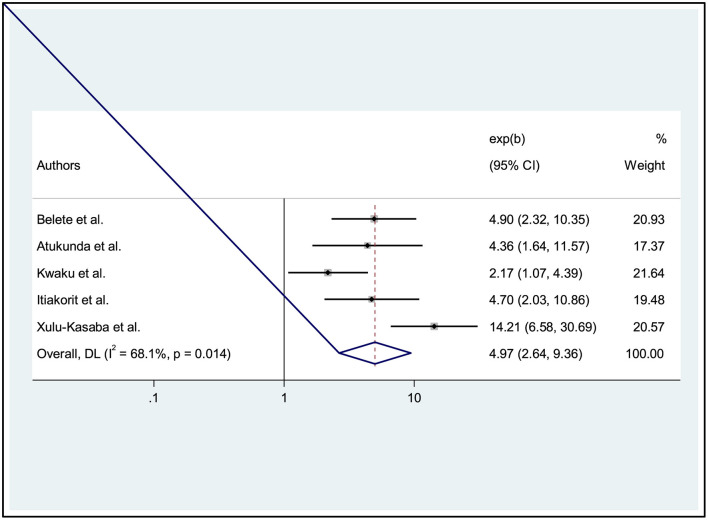
Forest plot showing the association between ocular protection practices and training on the welders.

Based on five studies (6, 25–27, 36), we determined statistical significance for the provision of training for welders in small-scale industries. The result showed that the combined effect of training among welders in small industries was significantly related to ocular protection practice in the workplace. Workers who had received occupational safety training were 4.97 times more likely to have practiced ocular protection than workers who had not received occupational safety training [AOR: 4.97, 95% CI: (2.64, 9.36)]. Moderate heterogeneity was observed across studies (I^2^ = 68.1%, *p* = 0.0.014), for this reason, we used a random effects model (Figure 4).

### The association between ocular protection practice and work experience of welders

**Figure 5:**
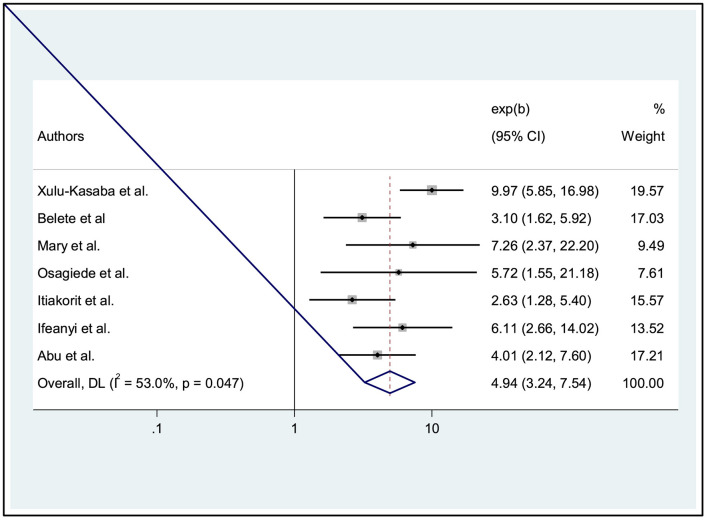
Forest plot showing the association between ocular protection practice and work experience of welders.

In addition, the influence of work experience on welders' ocular protection practices was examined through the analysis of seven studies (26, 27, 29–31, 35, 36). The results showed a significant association between welders' work experience and ocular protection practices. Specifically, those with work experience were found to be 4.94 times more likely to adopt ocular protection practices than the reference group (AOR: 4.94, 95% CI: 3.24, 7.54). A random effects model was used (I^2^ = 53.0%, *p* = 0.047) (Figure 5).

### The association between ocular protection practice and history of ocular injury

**Figure 6:**
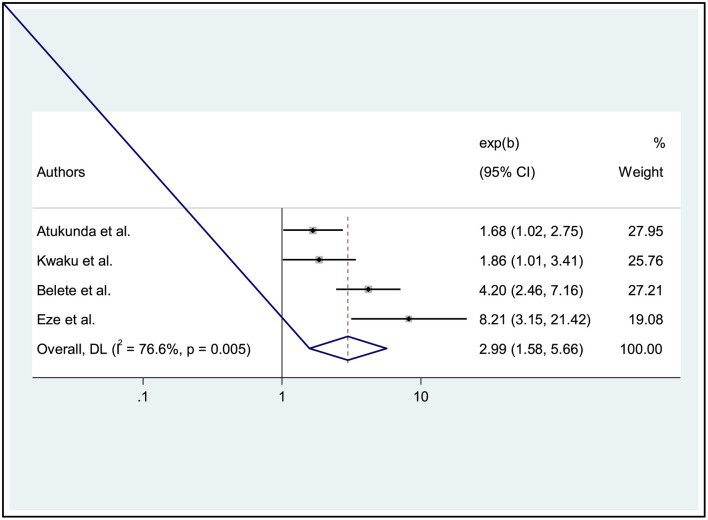
Forest plot showing the association between ocular protection practice and history of ocular injuries of welders.

Finally, four studies showed that a history of eye injury in workers was significantly associated with ocular protection practices (6, 25, 27, 28). Welders with a history of eye injuries in the past year were 2.99 times more likely to perform eye protection practices than reference subjects [AOR: 2.99, 95% CI: (1.58, 5.66)]. Since the heterogeneity was moderate, we used a random effects model (I^2^ = 76.6%, *p* = 0.005) (Figure 6).

## Discussions

This study sought to determine the pooled prevalence, pattern, and factors associated with ocular protection practices in small-scale welders in sub-Saharan Africa. Overall, the study found the pooled prevalence of reported ocular protection practice among welders in sub-Saharan Africa to be 53.71% (95% CI: 42.54, 64.88). This is similar to the results obtained in a study conducted in North India by Dahiya who found the prevalence of eye injury among welders to be 52.97% (38). However, the prevalence of this study finding was lower compared to 75.0% by Prabhu in South India (39), and 61.9% by Wang in Taiwan (40). According to Wang, the surge in industrialization was directly linked to the heightened occurrence of the phenomenon (40).

These differences may be attributed to occupational status, as our research included the prevalence of various occupational exposures. In contrast, the above studies focused exclusively on single occupation, which may have resulted in a decrease in the prevalence rate. On the other hand, social education and socioeconomic status are lower in sub-Saharan Africa, which may affect workers' practices in implementing preventive measures to protect against occupational problems, and differences in measurement of outcome variables, and sample size may also contribute to these differences.

Moreover, the prevalence of this study finding was higher compared to 31.1% by Praveena in Western Rajasthan (41), and 45.2% by Tenkate in Canada (42). Differences in study designs, inclusion criteria, data collection instruments, and sample sizes could explain the observed differences. Unlike previous studies that included workers from various small-scale industries such as wood, metal, and brick, the current study focused specifically on welders.

Regarding health and safety training, workers who had received OHS training were more likely to practice eye protection than workers who had not received OHS training. This might be because as workers have occupational health and safety training, they may have a piece of information on different ocular injuries when to use protective devices, and which specific type must be used to prevent ocular injuries. These will help them to use different protective measures to reduce their risk of having ocular injuries. This result agreed with those studies conducted in Western Rajasthan (41), and Northern Thailand (43).

In the present study, the work experience of workers per year was statistically significant concerning ocular protection practices. Workers who have no work experience were less likely to have ocular protection practices than those who had some work experience. This was consistent with a study done in Taiwan (40), Tenkate in Canada (42), and Sundar Budhathoki in Nepal (44). By improving welders' work experience, their awareness of welding-related eye hazards and compliance with the use of personal eye protection devices could be increased by attending various safety training courses.

Furthermore, the welder workers who have a history of ocular injuries in the past year were more likely to perform the practice of ocular protection than the workers who have not had a history of ocular injury. This finding was in line with those studies conducted by Zakrzewski in Canada (45), Australia (46), South Korea (47), and Limbu in Nepal (48). Because of the awareness of welding-related eye hazards and the use of personal eye protection devices.

## Strengths and limitations of the study

We conducted a thorough and methodical search of articles, ensuring that our selection criteria were clearly defined to minimize any potential bias in our study. However, it is important to acknowledge that there were certain limitations in our search process. Specifically, we may have overlooked some relevant literature as we only included articles written in English and certain databases were not included in our search. Furthermore, we also incorporated preprint articles that have not yet undergone peer review. It is worth noting that the findings from these studies may evolve in future. Additionally, it is important to consider the possibility of methodological biases that may be present in our included studies.

## Conclusions

The current study revealed that more than half of the study participants had good ocular protection practices in sub-Saharan Africa, which are significantly associated with on-the-job training, work experience of workers, and a history of ocular injury in the past years. This pooled study findings will serve as baseline data for further studies to generate inputs for occupational health care providers and policymakers to design evidence-based interventions to reduce the burden of occupational ocular injuries.

## Data availability statement

The original contributions presented in the study are included in the article/Supplementary material, further inquiries can be directed to the corresponding author.

## Author contributions

YA: Writing – review & editing, Writing – original draft, Visualization, Validation, Software, Resources, Methodology, Investigation, Formal analysis, Data curation, Conceptualization. NG: Data curation, Formal analysis, Writing – original draft, Supervision, Writing – review & editing, Conceptualization, Investigation, Software. KG: Writing – original draft, Visualization, Validation, Supervision, Software, Resources, Project administration, Methodology, Investigation, Formal analysis, Conceptualization.
