# Supplementary material for: Systematic review and meta-analysis on prevalence, pattern, and factors associated with ocular protection practices among welders in sub-Saharan Africa
Source: Front Public Health. 2024 Jun 17;12:1397578. doi: 10.3389/fpubh.2024.1397578 (PMC11215141; doi:10.3389/fpubh.2024.1397578)
Supplement: Supplementary file 2 [file Table_2.docx]

**Supplementary 2**. Quality assessment for the included Studies

| **Item** | **Clearly defined inclusion** | **Describe the study setting and participant** | **Valid and reliable exposure measurement** | **Objective and standard criteria for measurement** | **Identified confounder** | **Strategies to deal with confounders** | **Valid and reliable outcome measurement** | **Appropriate static analysis** | **No of ‘yes’ ‘** |
| --- | --- | --- | --- | --- | --- | --- | --- | --- | --- |
| Yego and Ragot et .al | Yes | Yes | No | Yes | Yes | No | Yes | Yes | 6/8=75 |
| Sithole et al. | Yes | Yes | Yes | Yes | No | No | Yes | Yes | 6/8=75 |
| Atukunda et al. | Yes | Yes | No | Yes | Yes | No | Yes | Yes | 6/8=75 |
| Kwaku et al. | Yes | Yes | No | Yes | Yes | Yes | Yes | Yes | 7/8=87.5 |
| Ajayi et al. | Yes | Yes | No | Yes | Yes | Yes | Yes | Yes | 7/8=87.5 |
| Itiakorit et al. | Yes | Yes | Yes | Yes | Yes | No | Yes | Yes | 7/8=87.5 |
| Belete et al. | Yes | Yes | No | Yes | Yes | Yes | Yes | Yes | 7/8=87.5 |
| Eze et al | Yes | Yes | Yes | Yes | No | No | Yes | Yes | 6/8=75 |
| Abu et al. | Yes | Yes | No | Yes | Yes | Yes | Yes | No | 6/8=75 |
| Mary et al. | Yes | Yes | Yes | Yes | No | Yes | Yes | No | 6/8=75 |
| Osagiede et al. | Yes | Yes | Yes | Yes | No | No | Yes | Yes | 6/8=75 |
| Ezinne et al. | Yes | yes | No | Yes | Yes | Yes | Yes | Yes | 7/8=87.5 |
| Belete et al. | Yes | Yes | Yes | No | Yes | No | Yes | Yes | 6/8=75 |
| Aziegbe et al. | Yes | Yes | Yes | Yes | Yes | Yes | Yes | No | 7/8=87.5 |
| Ifeanyi et al. | Yes | Yes | Yes | Yes | Yes | Yes | No | Yes | 7/8=87.5 |
| Xulu-Kasaba et al. | Yes | Yes | No | Yes | Yes | Yes | Yes | Yes | 7/8=87.5 |
| Rongo et al. | Yes | Yes | No | Yes | No | Yes | Yes | Yes | 6/8=75 |
